# The clinical characteristics of anemia in native adults living at different altitudes of the Tibetan Plateau

**DOI:** 10.1038/s41598-022-26868-7

**Published:** 2023-02-24

**Authors:** Jie Fang, Ran Li, Dongdong Ye, Liang Chen, Luo Bu Zhuo Ma, Yinyin Zhang, Jun Zhu, Xiaodong Gao, Pengpeng Xu, Yu Zheng, Xiaoyang Li, Jianhua You, Chuanhe Jiang, Kai Qing, Fei Yue, Junmin Li, Pu Bu Wang Dui, Kai Xue

**Affiliations:** 1 Department of Hematology, Shigatse People’s Hospital, Tibet Autonomous Region, China; 2 Shanghai Institute of Hematology, State Key Laboratory of Medical Genomics, National Research Center for Translational Medicine at Shanghai, Ruijin Hospital, Shanghai Jiao Tong University School of Medicine, Shanghai, China; 3 Department of Medical Oncology, Ruijin Hospital Wuxi Branch, Shanghai Jiao Tong University School of Medicine, Wuxi, China; 4 Department of Emergency Medicine, Xinhua Hospital, Shanghai Jiao Tong University School of Medicine, Shanghai, China

**Keywords:** Anaemia, Risk factors

## Abstract

To provide evidence-based medicine references for formulating prevention and control policies in plateau areas, we explore the characteristics of anemia patients in Tibet (the plateau areas of China), especially those located at an altitude above 4500 m. We collected clinical data from 379 Tibetan anemia patients over the age of 18 years. We found those female patients accounted for the majority of Tibetan anemia patients. Almost half of the anemia patients aged from 28 to 47 years. The percentage of severe anemia and extremely severe anemia was 45.4% and 2.4%, respectively. 88.7% of patients are engaged in agriculture and animal husbandry, and 81.5% of patients just graduated from primary school or below. The most common causes of anemia were nutritional anemia, especially iron-deficiency anemia. At high-altitude localities, folic acid-deficiency anemia needs more attention. Overall, this study showed that altitude influences the incidence, severity, and cause of anemia. Peasants and herdsmen, low education levels, young and middle-aged women, and nutrition status should be paid attention to in future anemia control.

## Introduction

Anemia is a kind of clinical syndrome in which the number and size of red blood cells, or the hemoglobin concentration, falls below an established cut-off value, consequently impairing the capacity of the blood to transport oxygen around the body. Among them, hemoglobin concentration is the most commonly used and reliable diagnosis criterion^1^. World Health Organization (WHO) reported that the anemia population in the world exceeds 2 billion now, accounting for 30% of the world population^2^. Anemia is still a common and frequently occurring disease. The incidence of anemia in plateau areas is relatively high, which is closely related to factors such as poor geographical environment, backward economic conditions, cultural beliefs, and a monotonous diet, which seriously endangers plateau residents' health^3–5^.

At present, there is a lack of a large amount of detailed data^6^ in plateau areas of China. In addition, the average altitude of Lhasa, Qinghai, Sichuan, Yunnan, and other Tibetan areas is low, and there is a lack of data^7^ above 4500 m altitude in such areas. Shigatse area covers an area of 182,000 square kilometers (about 2 areas of Jiangsu Province). The population is about 800,000, accounting for about 1/4 of the total population of Tibet. It has a diversity of altitudes (the highest is 8,848 m of Qomolangma, the lowest is 1600 m of Yadong, and the average is 3600 m). As it is located in the hinterland of Tibet, the hospitalized patients are mainly local Tibetan residents, which has a good regional representation.

To meet the national demand for targeted poverty alleviation, this retrospective study collected 379 hospitalized anemia patients over the age of 18 years who were admitted to the Hematology Department of Shigatse People's Hospital from November 2017 to November 2021. The demographics and clinical data were collected and the characteristics of the subjects, like the age, gender, altitude, education, occupation, and severity of anemia were analyzed. The study aimed to provide an evidence-based reference for making the follow-up targeted intervention measures to prevent and treat anemia.

## Methods

### Data acquisition

From November 2017 to November 2021, a total of 379 patients with anemia older than 18 were selected from the Hematology Inpatient Department at The Shigatse People's Hospital. Clinical data (age, gender, etc.) were collected and the details of baseline clinical characteristics were displayed in Table 1. The anemia data from the examination report of the blood routine from outpatients or inpatients. All subjects provided informed consent and the study is by the Helsinki Declaration.

**Table 1:** Characteristics of eligible individuals.

Parameters	Patients (%)
**Age**	
< = 60	306 (80.7)
> 60	73 (19.3)
**Gender**	
Male	51 (13.5)
Female	328 (86.5)
**Altitude**	
< = 3500	10 (2.6)
3500–4500	261 (68.9)
> 4500	108 (28.5)
**Education**	
Primary school and below	309 (81.5)
Junior high school	28 (7.4)
High school	11 (2.9)
Beyond high school	31 (8.2)
**Occupation**	
Herdsman	336 (88.7)
Non-herdsman	43 (11.3)
**Anemia**	
Mild	13 (3.4)
Moderate	185 (48.8)
Severe	172 (45.4)
Extremely severe	9 (2.4)

### Diagnosis criteria of anemia in Plateau

Anemia was defined as hemoglobin (HGB) < 120 g/L for an adult male, HGB < 110 g/L for an adult female (non-pregnant), and HGB < 100 g/L for pregnant women per Chinses criteria^8^. The severity of anemia was classified as very severe (if HGB value < 30 g/L), severe (if HGB value is 30–60 g/L), moderate (if HBG value is 60–90 g/L), and mild (if HGB value is 90–120 g/L for males and 90–110 g/L for females) according to the HGB thresholds^9^. The correction of diagnostic criteria for anemia is to eliminate the influence of altitude on the HGB level. In plateau areas above 3500 m, the diagnostic criteria for anemia are adjusted because HGB increases by about 4% for every 1000 m increase in altitude^10^.

### Statistical analysis

Statistical Package for Social Sciences (SPSS) version 26.0 was used to conduct all the statistical analyses of this study. The counting data were expressed by percentage or absolute value, and the ratio between the two groups was tested by the chi-square test. Differences were considered statistically significant when p values were less than 0.05 (*p* < 0.05). The data were marked as *p < 0.05, **p < 0.01, and ***p < 0.001.

### Ethics approval and consent to participate

All patients provided informed consent for sample collection. Protocols for peripheral blood sample handling and data analysis were approved by the Institutional Review Board from Shigatse People's Hospital.

## Results

### The distribution of anemia in Tibetan

We first focused on the differential distributions of anemia by age, gender, altitude, education, occupation, and severity of anemia (Table 1). We found that anemia was mainly distributed in populations older than 60 years (80.7%), females (86.5%), people living at altitudes from 3500 to 4500 m (68.9%), people with low education levels (primary school or below, 81.5%) and herdsman (88.7%). The severity of anemia was mainly moderate (48.8%) and severe (45.4%).

### The distribution of the degree of anemia in different subgroups

**Figure 1:**
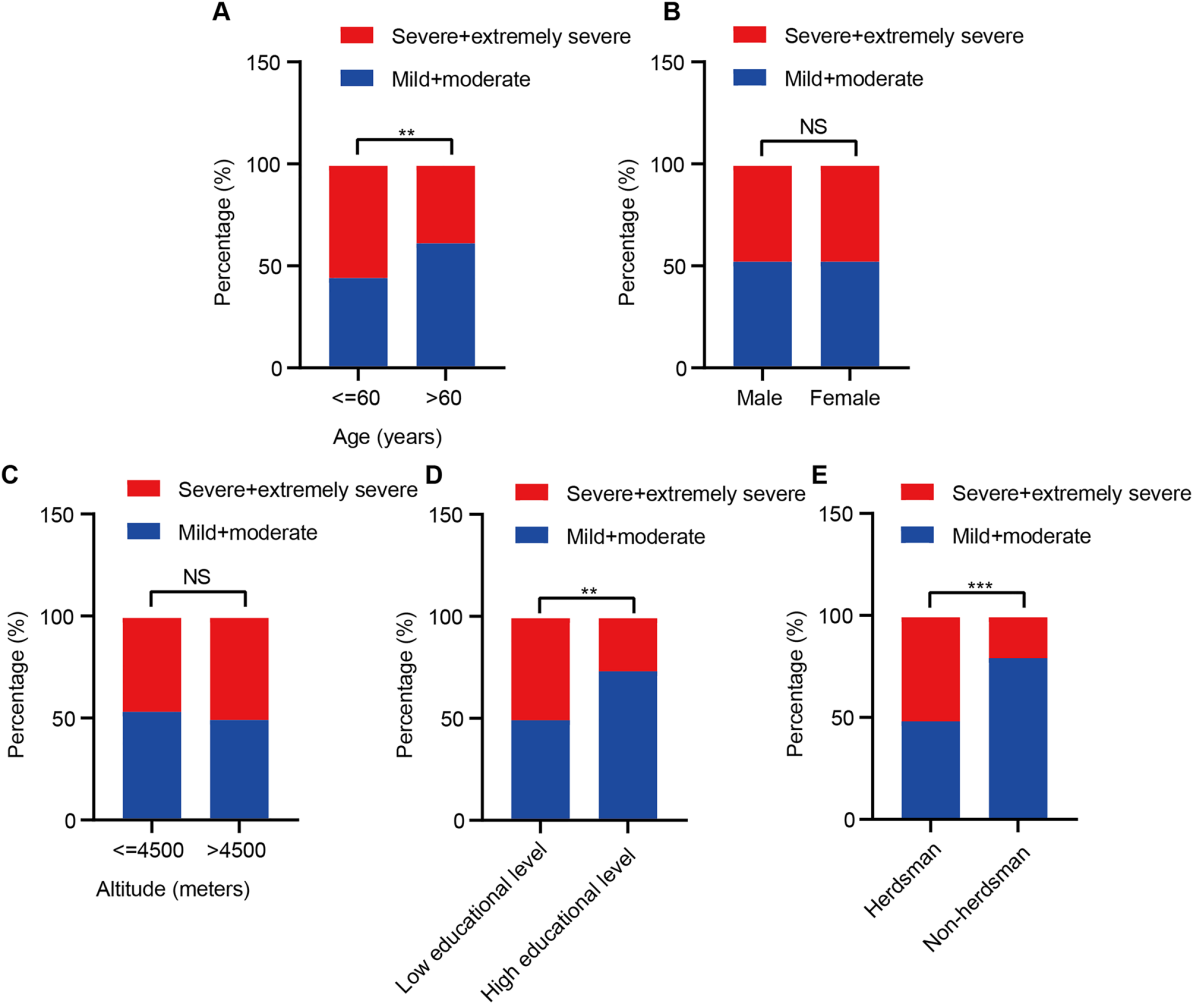
The distribution of the degree of anemia in different subgroups, including age (**A**), gender (**B**), altitudes (**C**), educational level (**D**), and occupation (**E**). Red represents the severe/extremely severe group, and blue represents the mild/moderate group. **p < 0.01; ***p < 0.001; NS, not significant.

We divided the severity of anemia into two groups, including the severe/extremely severe group, and the mild/moderate group. Patients who are younger (≤ 60 years) are more likely to be present in the severe/extremely severe anemia group. The differences were considered statistically significant (Fig. 1A). There was no significant difference between the two groups in terms of gender (Fig. 1B) or altitude (Fig. 1C). In addition, the proportion of people with low educational levels (junior high school and primary school or lower level, Fig. 1D) was significantly greater in the severe/extremely severe group. Similar phenomena were observed when the same analyses were performed between herdsmen or not (Fig. 1E). Detailed information on the severity of anemia in different subgroups were shown in Tables S1–S5.

### Causes of anemia in Tibetan

**Figure 2:**
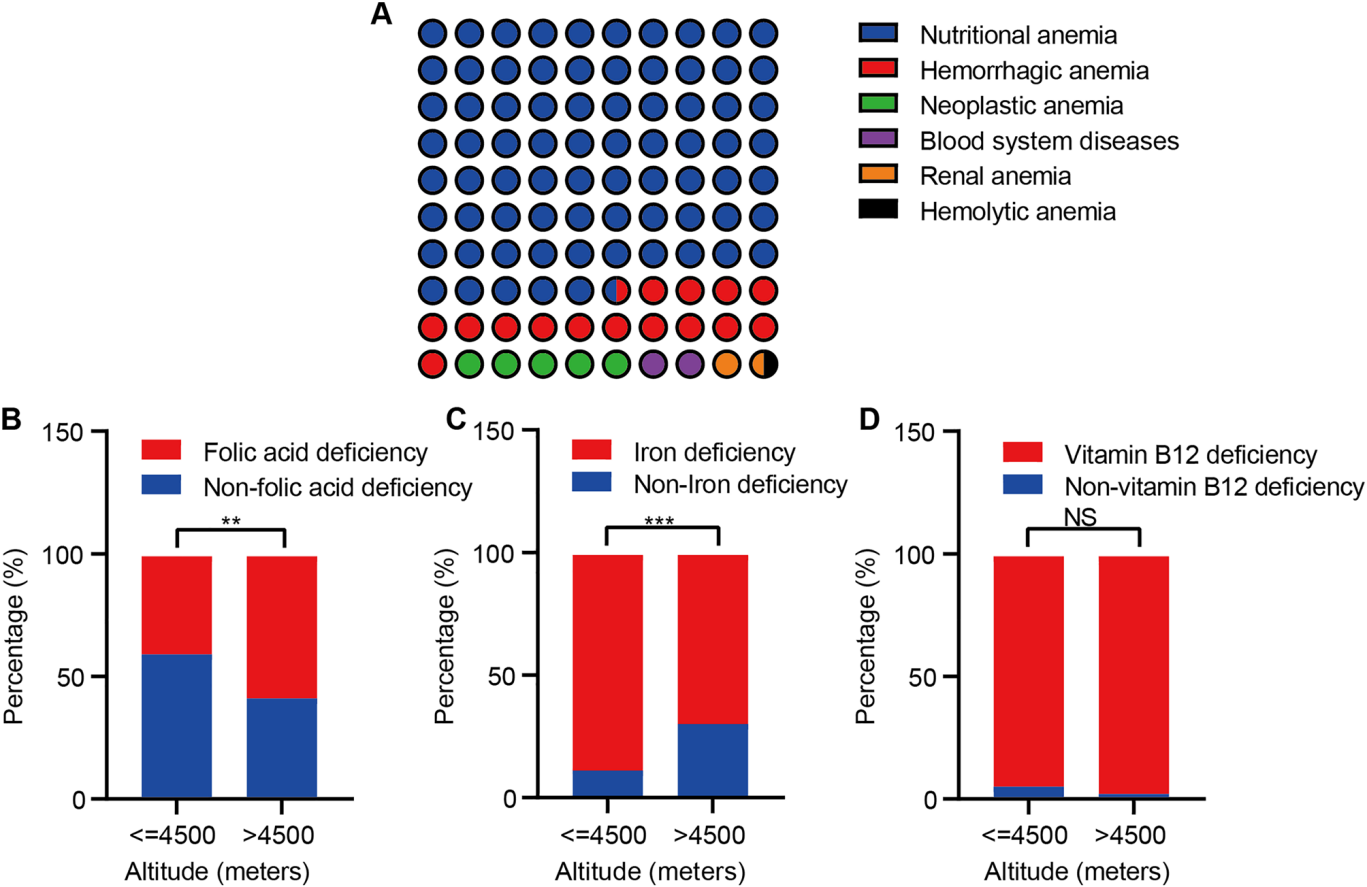
A displayed various causes of anemia in Tibetan. (**A**) The frequency distribution of different causes of anemia in Tibetan. (**B**–**D**) The distributions of folic acid, iron, and vitamin B12 deficiency at different altitudes. Each color from red to purple on the right side of image A represents a different cause of anemia. In images B, C, and D, red represents the deficiency of folic acid, iron, and vitamin B12, whereas blue indicates Nondeficiency. **p < 0.01; ***p < 0.001; NS, not significant.

We analyzed the cause of anemia in Tibetan and found that nutritional anemia (75.7%), hemorrhagic anemia (15.5%), and neoplastic anemia (4.8%) are the main types of anemia in Tibetan (Fig. 2A). Detailed information on different nutritional anemia types was shown in Table S6. Next, we analyzed the nutritional status of Tibetan at different altitudes. The results showed that the percentage of patients with folic acid deficiency was significantly higher at altitudes above 4500 m (Fig. 2B) while the iron deficiency proportion was lower (Fig. 2C). No significant differences were found among the vitamin B12 deficiency percentage at different altitudes (Fig. 2D). For hemorrhagic anemia, hysteromyoma, chronic gastrointestinal bleeding, and bleeding during delivery ratios at different altitudes, there was no significant difference. (Figure S1, Table S7).

## Discussion

In this study, all the participants enrolled were Tibetan from Shigatse, Tibet, China. We categorized anemia according to the severity or cause and finally figure out the unique regional characteristics of anemia in the plateau region by statistical methods. The results show that anemia patients mainly live at altitudes above 3500 m (96.5%), which are different from the plain areas in China (especially in the East). More than 47.8% of patients were severe or very severe anemic and female patients represent a significant proportion (86.5%). The proportion of men and women is further unbalanced. The proportion of young adults aged 28–47 years old, the main force of the labor force, is relatively high, about 47.5%. The majority of patients are peasants and herdsmen (88.7%), and most of them (81.5%) just graduated from primary school or below. Hence, much attention should be paid to the anemia problem in the plateau areas and appropriate interventions should be taken to prevent and treat anemia.

The incidence of anemia in women is significantly higher than that in men, which may be related to factors such as hemorrhage in women's physiological period, pregnancy, lactation, and internal hormones^11^. In addition, compared with the eastern plain area, the medical strength of gynecology and obstetrics is weak, and the socioeconomic development and cultural cognition level are relatively low^12^. Community and township hospitals must strengthen the construction of science popularization with local women as the core node. The township health centers should conduct regular physical examinations and screening, and carry out physical examinations regularly for young and middle-aged people (especially women of childbearing age and pregnancy), to timely find and solve potential disease hazards. The strength of obstetrics and gynecology in plateau areas, especially in higher altitude areas (> 4500 m), needs to be strengthened, The construction of medical association, training of obstetricians and gynecologists, remote consultation mechanism, two-way referral, etc. can be considered^13^.

According to the causes of anemia, further analysis was conducted. Nutritional anemia was the most common cause^14^, with 288 cases, accounting for 76% of the total number of anemia. It is caused by iron deficiency, folic acid, or vitamin B12 deficiency. The three substances mentioned above are important elements of life and are necessary for the synthesis of hemoglobin. However, the human body cannot synthesize folic acid and vitamin B12 by itself, and needs to be taken from food^15^.

If the supplement is insufficient, anemia is easy to occur^16^. Folic acid widely exists in plants and animals, such as fresh vegetables, fruits, meat, animal liver and kidney^17^, etc.; Vitamin B12 is mainly provided by animal foods, such as animal liver and kidney, meat, fish, eggs, and dairy products. Due to the harsh climate, convenient logistics, remote living areas, cultural beliefs, and other factors, the diet in plateau areas is mainly Zanba, milk, and butter, and the intake of fresh vegetables and fruits is less, resulting in a higher proportion of lack of folic acid. Especially in extremely high altitude areas (> 4500 m), folic acid deficiency deserves further attention. The incidence of folic acid deficiency is 57.9%, which is higher than 41.1% in patients at a lower altitude of 3500–4500 m (*p* < 0.05). Therefore, it is urgent to strengthen the publicity and education of nutrition knowledge among vulnerable populations, especially women of childbearing age, pregnancy, and lactation. Besides, we should pay more attention to the quality of diet, correcting partial eating, changing bad cooking habits, further optimizing logistics, developing nutrition tablets suitable for plateau areas, cultivating plateau green vegetables, improving medical awareness, and so on to prevent anemia^18^. In the early stage, the research group developed a nutritional butter tea with iron, folic acid, vitamin B12, and other ingredients to treat local patients with nutritional anemia, and achieved a better curative effect^14,19^.

Among the 379 patients with anemia, there were 58 patients with hemorrhagic anemia. Menorrhagia and more bleeding during childbirth due to uterine fibroids are common causes, accounting for 65.5% (35 patients) of all cases of hemorrhagic anemia. All the anemia patients lived in remote areas at an altitude above 3500 m and most of them were young or middle-aged female farmers and herdsmen. As mentioned above, the local health department should strengthen the medical power of gynecology and obstetrics and popularize the knowledge of gynecology and obstetrics health among relevant specific groups.

There were 18 cases of anemia caused by solid tumors, including 13 cases of digestive system tumors (including 6 cases of gastric cancer), 4 cases of bladder cancer, and 1 case of a tumor whose primary site was not found. All 6 cases of gastric cancer live at a high altitude of more than 3500 m. The literature suggests that the higher incidence of gastric cancer in plateau areas is related to the high infection rate of *Helicobacter pylori*^20^, heavy oil and salt diet^21^, alcoholism^22^, relatively backward medical conditions^23^, hypoxia affecting the repair of the gastric mucosal barrier and other factors^24^. Due to the reduction of gastric mucosal barrier capacity, some studies even suggest that the course of conventional anti-HP treatment needs to be further increased^25^ and the treatment intensity needs to be further confirmed by prospective clinical studies. Therefore, it should be advocated to actively carry out the early screening and prevention of *Helicobacter pylori* and gastric cancer.

Different from the eastern plain, there were relatively few malignant hematological diseases, a total of eight cases (2.1%)^26^. Including one case of aplastic anemia, 3 cases of myelodysplastic syndrome, and 4 cases of acute leukemia. Interestingly, the possible effects of hypoxia and low pressure on red blood cell life have been considered^27^. However, there are only 2 cases of hemolytic anemia. This may be related to the small population and low incidence rate of hematological malignancies in Shigatse, Tibet. However, the regional medical differences, like the development of the local medical system are relatively slow and some hematological diseases cannot be diagnosed due to the lack of relevant detection technologies were not excluded. In addition, the effect of the high-altitude environment on the incidence of benign and hematological diseases also needs further study^13^.

The purpose of this study was to explore the characteristics of Tibetan anemia patients in plateau areas of China and to provide evidence-based medicine references for formulating prevention and control policies. In short, anemia in plateau areas deserves further attention at present, and young and middle-aged women are the focus of anemia prevention and control in the future. The main types of anemia according to causes are nutritional anemia, hemorrhagic anemia, and cancer-related anemia (especially digestive system tumors). Higher altitude (> 3500 m) influences the occurrence, degree, and cause of anemia. It is suggested to further strengthen the publicity and education of anemia knowledge and reasonable dietary structure among Tibetan plateau people. On the other hand, the following specific measures can be: develop new food and agricultural technologies, strengthen the training of specialists in obstetrics and gynecology, and gastroenterology in remote areas, even the multiregional tour system of specialists and promote a variety of means of medical integration.

## Supplementary Information

Supplementary Information.

## Data Availability

All data generated or analysed during this study are included in this published article [and its supplementary information files].
