# Supplementary material for: The clinical characteristics of anemia in native adults living at different altitudes of the Tibetan Plateau
Source: Sci Rep. 2023 Feb 24;13:3208. doi: 10.1038/s41598-022-26868-7 (PMC9958083; doi:10.1038/s41598-022-26868-7)
Supplement: Supplementary file 1 — Supplementary Information. [file 41598_2022_26868_MOESM1_ESM.docx]

**Supplementary materials**


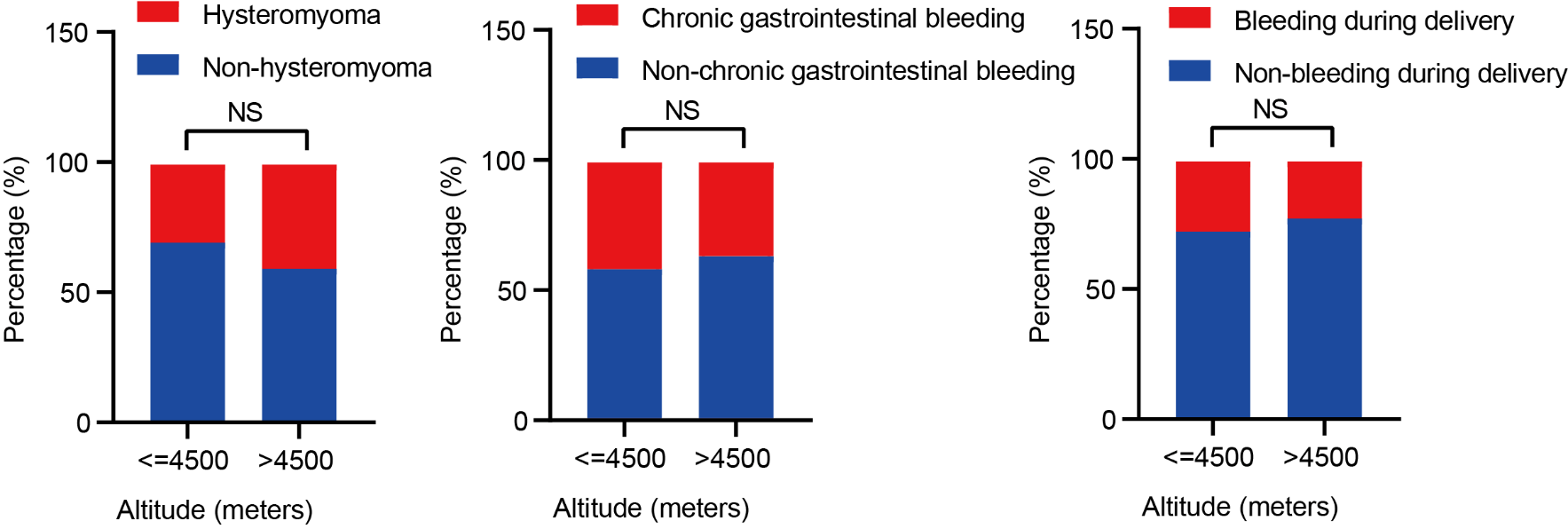


**Figure S1. The causes of hemorrhagic anemia at different altitudes**

| **Table S1. The degree distribution of anemia at different ages** | | | | | |
| --- | --- | --- | --- | --- | --- |
| **Age (years)** | **Extremely severe (%)** | **Severe (%)** | **Moderate (%)** | **Mild (%)** | **Total (%)** |
| **18-27** | 1 (1.6) | 30 (48.4) | 29 (46.8) | 2 (3.2) | 62 (16.4) |
| **28-37** | 0 (0) | 34 (43.6) | 38 (48.7) | 6 (7.7) | 78 (20.6) |
| **38-47** | 2 (2.0) | 37 (36.3) | 61 (59.8) | 2 (2.0) | 102 (26.9) |
| **48-57** | 2 (3.8) | 23 (43.4) | 26 (49.1) | 2 (3.8) | 53 (14.0) |
| **58-67** | 2 (4.3) | 24 (52.2) | 19 (41.3) | 1 (2.2) | 46 (12.1) |
| **≥68** | 1 (2.6) | 24 (63.2) | 12 (31.6) | 1 (2.6) | 38 (10.0) |

| **Table S2. The degree distribution of anemia in men and women** | | | | | | | | | | | |
| --- | --- | --- | --- | --- | --- | --- | --- | --- | --- | --- | --- |
| **Gender** | **Extremely severe (%)** | | **Severe (%)** | | **Moderate (%)** | | **Mild (%)** | | **Total (%)** | |  |
| **Male** | 1 (2.0) | | 23 (45.1) | | 24 (47.6) | | 3 (5.9) | | 51 (13.5) | |  |
| **Female** | 8 (2.4) | | 149 (45.4) | | 161 (49.1) | | 10 (3.0) | | 328 (86.5) | |  |
| **Table S3. The degree distribution of anemia at different altitudes** | | | | | | | | | | | |
| **Altitude (meters)** | | **Extremely severe (%)** | | **Severe (%)** | | **Moderate (%)** | | **Mild (%)** | | **Total (%)** | |
| **<=3500** | | 0 (0) | | 6 (60.0) | | 4 (40.0) | | 0 (0) | | 10 (2.6) | |
| **3500-4500** | | 6 (2.3) | | 114 (43.7) | | 131 (50.2) | | 10 (3.8) | | 261 (68.9) | |
| **>4500** | | 3 (2.8) | | 52 (48.1) | | 50 (46.3) | | 3 (2.8) | | 108 (28.5) | |

| **Table S4. The degree distribution of anemia at different education level** | | | | | |
| --- | --- | --- | --- | --- | --- |
| Education | **Extremely severe (%)** | **Severe (%)** | **Moderate (%)** | **Mild (%)** | **Total (%)** |
| Primary school and below | 7 | 144 | 150 | 8 | 309 |
| Junior high school | 1 | 18 | 9 | 0 | 28 |
| High school | 0 | 5 | 5 | 1 | 11 |
| Beyond high school | 0 | 6 | 21 | 4 | 31 |

| **Table S5. The degree distribution of anemia in different occupations** | | | | | |
| --- | --- | --- | --- | --- | --- |
| **Gender** | **Extremely severe (%)** | **Severe (%)** | **Moderate (%)** | **Mild (%)** | **Total (%)** |
| Herdsman | 8 | 164 | 157 | 7 | 336 |
| Non-herdsman | 0 | 9 | 28 | 6 | 43 |

| **Table S6. The status of nutritional anemia at different altitudes** | | | | | | |  |  |
| --- | --- | --- | --- | --- | --- | --- | --- | --- |
| **Altitude (meters)** | **Iron deficiency** | **Folic acid deficiency** | **Vitamin B12 deficiency** | **Iron and Folic acid deficiency** | **Iron and Vitamin B12 deficiency** | **Folic acid and Vitamin B12 deficiency** | **Iron, Folic acid and Vitamin B12 deficiency** | **Total (%)** |
| **<=3500** | 4 (40.0) | 0 (0) | 0 (0) | 6 (60.0) | 0 (0) | 0 (0) | 0 (0) | 10 (3.5) |
| **3500-4500** | 113 (56.2) | 22 (10.9) | 2 (1.0) | 57 (28.4) | 5 (2.5) | 4 (2.0) | 1 (0.5) | 201 (70.8) |
| **>4500** | 28 (38.4) | 22 (30.1) | 0 (0) | 21 (28.8) | 2 (2.7) | 0 (0) | 0 (0) | 73 (25.7) |

| **Table S7. The causes of hemorrhagic anemia at different altitudes** | | | |  |
| --- | --- | --- | --- | --- |
| **Altitude (meters)** | **Hysteromyoma** | **Chronic gastrointestinal bleeding** | **Bleeding during delivery** | **Total (%)** |
| **<=3500** | 0 (0) | 0 (0) | 0 (0) | 0 (0) |
| **3500-4500** | 11 (30.6) | 15 (41.7) | 10 (27.8) | 36 (62.1) |
| **>4500** | 9 (40.9) | 8 (36.4) | 5 (22.7) | 22 (37.9) |
